# B-Vitamin Intake and Biomarker Status in Relation to Cognitive Decline in Healthy Older Adults in a 4-Year Follow-Up Study

**DOI:** 10.3390/nu9010053

**Published:** 2017-01-10

**Authors:** Catherine F. Hughes, Mary Ward, Fergal Tracey, Leane Hoey, Anne M. Molloy, Kristina Pentieva, Helene McNulty

**Affiliations:** 1 Northern Ireland Centre for Food and Health, Ulster University, Cromore Road, Coleraine BT52 1SA, Northern Ireland, UK; mw.ward@ulster.ac.uk (M.W.); l.hoey@ulster.ac.uk (L.H.); k.pentieva@ulster.ac.uk (K.P.); h.mcnulty@ulster.ac.uk (H.M.); 2 Causeway Hospital, Northern Health and Social Care Trust, Coleraine BT52 1HS, Northern Ireland, UK; Fergal.Tracey@northerntrust.hscni.net; 3 School of Medicine, Trinity College Dublin, Dublin 2, Ireland; AMOLLOY@tcd.ie

**Keywords:** one-carbon metabolism, B-vitamin biomarkers, dietary intakes, vitamin B6, pyridoxal-5-phosphate (PLP), cognition, ageing

## Abstract

Advancing age can be associated with an increase in cognitive dysfunction, a spectrum of disability that ranges in severity from mild cognitive impairment to dementia. Folate and the other B-vitamins involved in one-carbon metabolism are associated with cognition in ageing but the evidence is not entirely clear. The hypothesis addressed in this study was that lower dietary intake or biomarker status of folate and/or the metabolically related B-vitamins would be associated with a greater than expected rate of cognitive decline over a 4-year follow-up period in healthy older adults. Participants (aged 60–88 years; *n* = 155) who had been previously screened for cognitive function were reassessed four years after initial investigation using the Mini-Mental State Examination (MMSE). At the 4-year follow-up assessment when participants were aged 73.4 ± 7.1 years, mean cognitive MMSE scores had declined from 29.1 ± 1.3 at baseline to 27.5 ± 2.4 (*p* < 0.001), but some 27% of participants showed a greater than expected rate of cognitive decline (i.e., decrease in MMSE > 0.56 points per year). Lower vitamin B6 status, as measured using pyridoxal-5-phosphate (PLP; <43 nmol/L) was associated with a 3.5 times higher risk of accelerated cognitive decline, after adjustment for age and baseline MMSE score (OR, 3.48; 95% CI, 1.58 to 7.63; *p* < 0.05). Correspondingly, lower dietary intake (0.9–1.4 mg/day) of vitamin B6 was also associated with a greater rate of cognitive decline (OR, 4.22; 95% CI, 1.28–13.90; *p* < 0.05). No significant relationships of dietary intake or biomarker status with cognitive decline were observed for the other B-vitamins. In conclusion, lower dietary and biomarker status of vitamin B6 at baseline predicted a greater than expected rate of cognitive decline over a 4-year period in healthy older adults. Vitamin B6 may be an important protective factor in helping maintain cognitive health in ageing.

## 1. Introduction

Advancing age can be associated with an increase in cognitive dysfunction, a spectrum of disability that ranges in severity from normal age-related changes through mild cognitive impairment (MCI) to dementia; with the latter defined as a progressive decline in memory, thinking, language and judgment that is sufficient to impair activities of daily living [1]. An estimated 50% of those diagnosed with MCI will go onto develop dementia within 5 years of diagnosis [2]. Globally, it is estimated that 48 million people are currently suffering from dementia and the figures are predicted to triple by 2050 [3]. Dementia is a leading cause of disability, dependency and decreased quality of life among older people [3] and presents many social, economic and health care challenges that will continue to increase with an ageing population. Therefore, the identification of strategies to prevent or delay the onset of dementia has become a major global public health priority.

A number of nutritional and lifestyle factors have emerged as potential modifiable risk factors for cognitive decline in ageing [4]. In particular, there is considerable epidemiological evidence to suggest that sub-optimal status of folate, the related B-vitamins, and/or elevated concentrations of the metabolite homocysteine, contribute to cognitive dysfunction [5,6,7,8,9,10] and to a greater rate of cognitive decline in ageing [11,12,13,14]. Elevated plasma homocysteine and lower folate have been most consistently associated with cognitive dysfunction in ageing [6,8]. There is also evidence to support a role for vitamin B12 [15,16] and to a lesser extent vitamin B6, although the latter has been far less extensively investigated [9,17]. There is also some evidence in the form of randomised controlled trials to show beneficial effects of B-vitamin supplementation on cognition in ageing [18,19,20]. A number of other trials have failed to detect significant benefits [21,22,23] with recent meta-analyses concluding that there was no beneficial effect of B-vitamin supplementation on cognition [24,25]. However, a number of these trials may have been too short in duration; conducted in healthy individuals, patients with severe dementia; or in those with optimal B-vitamin status and so unlikely to benefit from vitamin supplementation [26]. The strongest evidence to date of a causal relationship between B-vitamins and cognition comes from the Homocysteine and B-vitamin in Cognitive Impairment (VITACOG) study. This study showed that combined B-vitamin supplementation for two years had beneficial effects on cognitive performance in participants with MCI and elevated plasma homocysteine concentrations [20]. More importantly, it also demonstrated that B-vitamin supplementation reduced the rate of brain atrophy by 30% as measured using MRI [27]. A subsequent report from the VITACOG investigators reported that the atrophy occurred in grey matter areas of the brain which are particularly vulnerable to Alzheimer’s disease [28].

The intervention doses administered in VITACOG were well in excess of recommended dietary intakes and therefore whilst the VITACOG papers provide powerful evidence of a role for folate, vitamin B12, and/or vitamin B6 in cognition, the relevance of these results to nutrition, and thus prevention of cognitive dysfunction in ageing is unclear. Furthermore, epidemiological research generally in this area has predominantly focused on plasma homocysteine, folate and vitamin B12; most studies have overlooked vitamin B6 and all have ignored the role of vitamin B2. Consequently, the influence of all the relevant B-vitamins involved in one-carbon metabolism on cognition is not fully understood. Therefore, the aim of this study was to investigate whether lower dietary intake or biomarker status of B-vitamins (folate, vitamin B12, vitamin B6 or riboflavin) at baseline was associated with a greater rate of cognitive decline over a 4-year follow-up period in healthy older adults.

## 2. Materials and Methods

### 2.1. Participant Recruitment and Study Design

Potential participants were identified from our records of a previous cross sectional study funded by the UK Food Standards Agency investigating B-vitamin dietary intakes and biomarker status in the healthy younger and older adults in Northern Ireland as previously described [29]. Healthy participants (*n* = 662; aged ≥18 years) were recruited to the original study and as part of the protocol, those aged ≥60 years completed a cognitive function test (Folstein’s Mini Mental State Examination MMSE; [30], the purpose of the original assessment was to ensure that the ability of participants to accurately recall food intake was not compromised. The current study involved the re-examination (4 years after initial screening) of those aged ≥60 years (*n* = 255). The exclusion criteria for the original study were: those with vitamin B12 deficiency (serum vitamin B12 < 111 pmol/L); self-reported history of cardiovascular, gastrointestinal, hepatic, renal, or haematological disease; use of medications that interfere with B-vitamin metabolism; taking supplements containing B-vitamins; having visited a country with a mandatory fortification policy for a period ≥2 weeks in the previous 6 months; plasma creatinine concentrations >130 µmol/L (generally indicative of renal impairment); and a score of <25 on the MMSE (indicative of cognitive impairment). Ethical approval was granted by the University of Ulster Research Ethics Committee (UUREC; Ref UUREC/07/005) and all participants provided written informed consent.

### 2.2. Cognitive Assessment

Cognitive function was assessed at baseline and at follow-up (between 3.5 to 4 years from initial screening for each participant) using the MMSE [30], one of the most widely used cognitive screening tools in a clinical setting. It is a global test of cognitive function and assesses the domains of orientation, registration, attention and concentration, recall and language. Overall the maximum score achievable is 30, with a score <25 indicating a possibility of cognitive impairment and a score <20 dementia [30].

### 2.3. Dietary and Lifestyle Assessment

Dietary intake was assessed using a 4-day food diary (for 4 consecutive days, including Saturday and Sunday, to account for the known variation in day-to-day intake) in combination with a food frequency questionnaire. This combined dietary method as described previously has been validated at this centre for the assessment of the four relevant B-vitamins against each of their blood biomarkers [29]. The food frequency questionnaire requested participants to state the frequency of consumption for food groups or specific branded products fortified with B-vitamins (e.g., ready-to-eat breakfast cereals, bars, breads and margarines). Participants provided details on brand names of the products consumed so that the fortification profile of any new foods could be established. By combining the 2 dietary collection methods, we were able to estimate dietary intakes of the relevant B-vitamins from both natural food sources and from fortified foods. Each participant received oral and written instructions on how to complete the food diary and food-frequency questionnaire. Any queries or discrepancies between the 2 dietary records were discussed with the participant and were clarified within 1 week of collection to enhance the accuracy of information on usual food intakes. Food portion sizes were estimated by the participant by using household measures and were later quantified by using published food portion size data [31]. The food-composition database WEIGHED INTAKE SOFTWARE PACKAGE (WISP, version 3; Tinuviel Software, Anglesey, UK) was used to calculate mean daily energy and B-vitamin intakes. This database has been customised at our centre to enable natural food folate to be distinguished from folic acid added to foods by manufacturers, and this allows the estimation of dietary folate equivalents (DFE; [29]).

A health and lifestyle questionnaire was used to obtain information on medical history including depression, smoking, use of alcohol and medication, and educational attainment. Height (m) and weight (kg) were measured at baseline and body mass index (kg/m^2^) was calculated.

### 2.4. Laboratory Analysis

All participants provided a fasting 30 mL blood sample at baseline. Sample preparation and fractionation were performed within 4 h of blood collection, and blood aliquots were stored at −80 °C until batch analysis. Plasma homocysteine was measured by fluorescence polarization immunoassay using the Abbot Imx analyser [32]. Red blood cell folate was measured by microbiological assay using *Lactobacillus casei* [33]. Vitamin B12 status was determined using a number of biomarkers; the direct measures were serum total vitamin B12 by microbiological assay using *Lactobacillus leichmanni* [34] and serum holoTC (the metabolically active fraction of vitamin B12) by microparticle enzyme immunoassay (AxSym Active-B12; Axis-Shield, Heidelberg, Germany); the functional biomarker serum methylmalonic acid (MMA) by gas chromatography mass spectrometry using methylchloroformate derivatization at University of Bergen, Norway. Plasma vitamin B6 (PLP) was measured by reversed phase, high performance liquid chromatography with fluorescence detection [35]. Riboflavin status was assessed using the erythrocyte glutathione reductase activation (EGRAC) where the ratio of FAD stimulated to unstimulated enzyme activity is calculated; higher EGRAC values indicate lower riboflavin status, and sub-optimal riboflavin status is generally recognised as a coefficient ≥1.3 [36]. The methylenetetrahydrofolate reductase (MTHFR) 677C→T polymorphism was identified by polymerase chain reaction amplification followed by *HinF1* restriction digestion [37]. Plasma creatinine was measured using a standard spectrophotometric assay with use of a chemistry analyzer (Hitachi; Roche Diagnostics Corporation, Indianapolis, IN, USA). Additionally, pepsinogen I and pepsinogen II were measured as markers of gastric atrophy by enzyme-linked immunosorbent assay (Biohit, Helsinki, Finland); a ratio of pepsinogen I:II < 3 is indicative of atrophic gastritis. All samples were analysed blind and duplicated. Quality controls were provided by repeated analysis of pooled samples.

### 2.5. Statistical Analysis

All statistical analysis was performed using SPSS software (version 22; SPSS UK Ltd., Chersey, UK). Prior to analysis, tests for normality were conducted and data were log transformed where appropriate. Differences at baseline and follow-up were assessed using a paired t-test for continuous data and chi-squared test for categorical data. Correlations between dietary intakes and corresponding blood biomarkers were calculated using Pearson’s correlation coefficient (r). Annual cognitive decline was calculated (baseline MMSE−follow-up MMSE)/(duration of follow-up) on an individual basis for each participant, accelerated cognitive decline was defined as a decrease in MMSE > 0.56 points per year [38]. Binary logistic regression analysis was used to assess health and lifestyle predictors of cognitive decline. The impact of B-vitamin dietary intake and blood biomarker status as predictors of cognitive decline was assessed using binary logistic regression after controlling for significant predictors of cognitive decline (age and baseline MMSE).

## 3. Results

**Figure 1:**
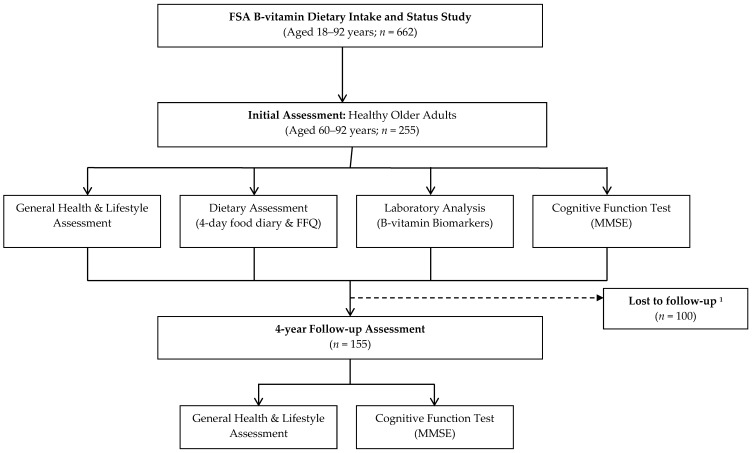
Study Design and flow of participants through the study. Abbreviations: FSA, Food Standards Agency; FFQ, Food Frequency Questionnaire; MMSE, Mini Mental State Examination; ^1^ Failed to meet inclusion criteria at follow-up assessment *n* = 26; declined to participate *n* = 43; deceased *n* = 4; non contactable *n* = 21; participation in other research *n* = 6.

Of the 662 healthy volunteers, 255 ≥ 60 years were identified as potential participants and of these 155 were available to participate in the follow-up assessment (Figure 1). Only those that participated at both timepoints are presented in this paper; those lost to follow-up were older and had significantly lower vitamin B12 status (Appendix A).

The characteristics of participants at initial screening are shown in Table 1. Participants had a mean age of 70 years, were predominantly female, well-educated and had a low rate of depression. The majority of participants were regular consumers of foods fortified with B-vitamins (75%). Dietary intakes compared favourably with current UK dietary recommendations [39] as reflected in good overall B-vitamin biomarker status. As a result of the exclusion criteria, no participant was deficient in vitamin B12, however some 3% were identified as deficient in folate and 11% deficient in vitamin B6. Gastric function was assessed by using pepsinogen I:II ratio, 12% had evidence of atrophic gastritis (pepsinogen I:II ratio < 3; data not shown).

**Figure 2:**
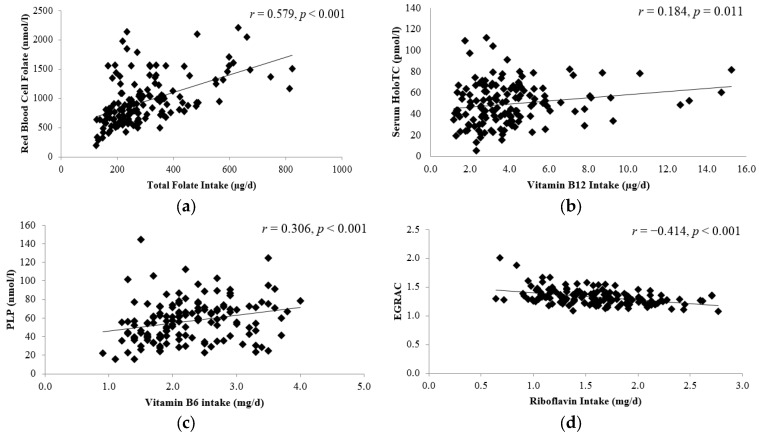
Relationship between dietary intake and biomarker status of B-vitamins at baseline (*n* = 148): (**a**) association between red blood cell folate and total folate intake; (**b**) association between holoTC and vitamin B12 intake; (**c**) association between pyridoxal-5-phosphate and vitamin B6 (**d**) association between EGRAC and riboflavin intake. Correlations were calculated using Pearson’s correlation coefficients (*r*). *p* < 0.05 was considered significant. HoloTC, holo-transcobalamin; PLP, Pyridoxal-5-phosphate—a measure of vitamin B6 status; EGRAC: erythrocyte glutathione reductase activation coefficient, a functional indicator of riboflavin status. The change in cognitive function score, as measured using MMSE is shown in Table 2. Over the 4-year follow-up period, a significant decline in the mean MMSE by almost 2 points was observed; the scores for each component of the MMSE (i.e., orientation, attention, recall, total verbal and language) also declined significantly, with the exception of registration. Whilst all participants had a MMSE score within the normal range at baseline (i.e., according to the inclusion criteria), 12% had a score indicative of mild cognitive impairment (MMSE range 18–24) at the time of follow-up. Overall, the average decrease in MMSE score per year was 0.42 ± 0.56; but some participants 42 (27%) had a greater than expected rate of cognitive decline (i.e., decrease in MMSE score > 0.56 points per year; [38].

The relationship between dietary intakes and corresponding blood biomarker concentrations were examined for each B-vitamin of interest (Figure 2). Dietary intakes for total folate, vitamin B6 and riboflavin were each significantly correlated with the corresponding blood biomarker concentration. Of note, vitamin B12 intake was significantly correlated with serum holoTC but not serum total vitamin B12, the more typically measured biomarker (*r* = 0.134, *p* = 0.104; data not shown). 

The influence of several lifestyle factors, B-vitamin dietary intake and B-vitamin biomarker status, as determinants of rate of cognitive decline are shown in Table 3. Of the general health and lifestyle factors examined only age and baseline MMSE score were predictive of cognitive decline. In addition, after adjustment for age and baseline MMSE score, no associations were observed between disease history (CVD, diabetes and gastrointestinal; data not shown) or medication use with the exception of use of analgesic medication (*p* = 0.035; data not shown). Vitamin B6 was found to be the only B-vitamin that was predictive of cognitive decline. After adjustment for age and baseline MMSE score, individuals with lower vitamin B6 biomarker status (PLP range ≤ 43.3 nmol/L; *p* = 0.002) or lower dietary B6 intakes (0.9–1.4 mg/day; *p* = 0.018) were at a 3.5–4 fold greater risk of cognitive decline. None of the other B vitamins or plasma homocysteine concentrations were associated with the risk of cognitive decline in this cohort.

## 4. Discussion

This study in healthy older adults, initially with normal cognitive performance, indicates that vitamin B6 is an important predictor of cognitive decline in ageing. Lower dietary intake and biomarker status of vitamin B6 were associated with a greater rate of cognitive decline over a subsequent 4 years follow-up period. No significant association of dietary intake or biomarker status with cognitive decline were observed for the other B-vitamins (folate, vitamin B12 and riboflavin). To our knowledge, this is the first longitudinal study to consider the impact of both dietary intake and biomarker status of all four relevant B-vitamins involved in one-carbon metabolism on cognitive health in ageing.

Whilst the influence of vitamin B6 on cognition has not been as fully investigated as folate and vitamin B12 a number of studies have reported observations consistent with the current study. Our results showed that participants with lower status of vitamin B6 (PLP; the measure of active vitamin B6) at baseline were 3.5 times more likely to have a greater rate of cognitive decline over a 4 years follow-up period. Furthermore, the association between vitamin B6 and cognitive decline was not confined to those with clinical deficiency, lower status included individuals in both the deficient (PLP < 30 nmol/L) and sufficient range (PLP 30–43 nmol/L) which would suggest that optimal vitamin B6 may be important for cognitive health in ageing. Consistent with the biomarker data, those with lower dietary intakes of vitamin B6 at baseline were 4 times more likely to have a greater rate of cognitive decline over the 4 years time period. Our results are in good agreement with findings from other studies, low vitamin B6 status (PLP < 46 nmol/L) and corresponding dietary intakes were previously associated with cognitive decline over a 3 years period in the Veteran Affairs Normative Ageing Study [40]. There is also evidence from several cross-sectional studies to support an association between low vitamin B6 and cognitive dysfunction [9,17,41,42] and Alzheimer’s disease [43,44]. Furthermore, vitamin B6 status was associated with cognitive performance in high functioning older adults at baseline, though not with cognitive decline over the 7 years follow-up period in the MacArthur study of Successful Ageing [8]. Certain other studies have failed to detect any significant association between vitamin B6 and cognitive function, however, these studies have relied on dietary intake measures alone with no corresponding measurement of blood biomarker status [45,46,47]. Few RCTs have investigated the independent effect of vitamin B6 on cognitive function and only one very early study reported beneficial effects of vitamin B6 supplementation on memory [19]. Subsequent RCTs have investigated the effect of vitamin B6 in combination with folate and vitamin B12, with some studies reporting beneficial effects on cognitive function however the independent effect of vitamin B6 cannot be determined [18,20].

Whilst elevated plasma homocysteine, low folate and, to a lesser extent, vitamin B12 status have been frequently associated with cognitive decline [11,12,13,14,15,48] there was no evidence of significant associations for these biomarkers in the current study. A number of other studies have reported similar findings [49,50,51,52]. The findings in the current study may be explained to some degree by the fact that vitamin B6 seemed to be the limiting nutrient within the cohort. There was a greater incidence of deficiency of vitamin B6 (11% clinical deficiency) compared with folate (3%) or vitamin B12 (0%). Also, the lack of a significant association between cognitive decline and plasma homocysteine concentration is almost certainly is a reflection of the low prevalence of folate deficiency. Furthermore, the concept that the association between B-vitamin status and cognition is determined by the limiting nutrient within that population group is further supported by evidence from published RCTs. One trial of healthy older adults in New Zealand reported no benefit of combined B-vitamin supplementation on cognitive function [21], whereas another similar study from the Netherlands showed that supplementation with folic acid significantly improved cognitive performance [18]. A notable difference between these two studies was that baseline folate status tended to be far lower in the Dutch trial, suggesting that the cognitive benefit related to the correction of sub-optimal B-vitamin status whereas additional B-vitamins to an already optimal population may have no beneficial effect.

The mechanism linking vitamin B6 with cognitive health in this and other studies in ageing is not clear however, it is biologically plausible given the widespread functions of vitamin B6 within the brain and nervous system [53,54]. Vitamin B6 has a crucial role in the synthesis of a variety of neurotransmitters including dopamine and serotonin [55] and can act as a potent antioxidant [56,57]. In addition, higher vitamin B6 intakes have been associated with greater grey matter volume [58] and combined B-vitamin supplementation (including vitamin B6) has been shown to slow brain atrophy, an important feature cognitive dysfunction [27].

The current study has a number of strengths and limitations that merit comment. To our knowledge, this is the first longitudinal study to investigate the association between cognitive decline and all relevant B-vitamins along with their corresponding dietary intakes. The MMSE is the most widely used screening tools for cognitive dysfunction and although it has been criticised for lacking sensitivity, few previous studies have used it to measure cognitive change in a healthy older population. However, a meta-analysis reported a mean decline in MMSE of between 0.16 and 0.56 points per year in cognitively healthy people which compares favourably to the overall rate of decline observed in this study (mean 0.39 points per year) [38]. In addition, the rate of decline observed in this study was identical to that observed in the Rotterdam Study of community dwelling older adults free from cognitive impairment [59]. While the use of the MMSE may be perceived as a limiting factor in the current study, it could be argued that its use would only attenuate the associations observed and that the use of more sensitive tools would have, if anything, detected more subtle differences thus strengthening the results. Another well-recognised limitation of longitudinal studies of this kind is that individuals with the greatest decline in cognitive function are more likely to be lost to follow-up [60]. Indeed, in this study the non-participants were more likely to be older but any non-response bias would ultimately underestimate the associations between baseline B-vitamin status and cognitive decline and this could not have influenced the current findings.

## 5. Conclusions

In conclusion, vitamin B6 may be an important (often overlooked) protective factor in helping maintain cognitive health in ageing, especially in a folate and vitamin B12 replete population. Lower vitamin B6 status (as assessed by both dietary intake and biomarker status) at baseline predicted a greater than expected rate of cognitive decline over a 4-year period in healthy free living older adults. These findings are important because optimising vitamin B6 status in older people, through the use of fortified foods or supplements, may have a positive impact on cognition in ageing. Further research in this area in the form of well-designed randomised controlled trials targeted at populations with sub-optimal status are required in order to confirm a cause and effect relationship between B-vitamin status and cognitive health in ageing.

## Figures and Tables

**Table 1:** General characteristics of healthy older adults at initial investigation (*n* = 155).

	Participants	Reference Range
Age (years)	69.5 (7.3)	
Male *n* (%)	60 (39)	
BMI (kg/m^2^)	27.5 (4.2)	20–25
Smokers *n* (%)	6 (4)	
3rd Level Education *n* (%)	48 (31)	
Depression *n* (%) *	11 (7)	
Cognitive Function (MMSE)	29.1 (1.3)	≤25
**B-Vitamin Dietary Intakes ^†^**		
Energy (MJ/day)	7.621 (1.789)	9.71 (M); 7.96 (F)
Total Folate (µg/day)	303 (141)	200
Vitamin B12 (µg/day)	4.0 (2.4)	1.5
Vitamin B6 (mg/day)	2.3 (0.7)	1.4 (M); 1.2 (F)
Riboflavin (mg/day)	1.6 (0.4)	1.3 (M); 1.1 (F)
Fortified Food Consumer *n* (%) ^‡^	116 (75)	
**B-Vitamin Biomarker Status** ^§^		
Red Blood Cell Folate (nmol/L)	954 (410)	340–2270
Serum total B12 (pmol/L)	282 (106)	111–740
Serum HoloTC (pmol/L)	50.8 (24.3)	40–200
Serum MMA (µmol/L)	0.24 (0.19	≤0.36
Vitamin B6 (Plasma PLP; nmol/L)	58.4 (25.8)	20–121
Riboflavin (EGRAC)	1.33 (0.14)	≤1.3
Plasma total homocysteine (µmol/L)	12.0 (3.7)	<10

Data presented as mean (SD) unless otherwise indicated. * History of depression was self-reported. ^†^ Reference ranges for dietary intakes based on reference nutrient intake values (RNIs) for 50+ years except for energy where the estimated energy requirements (EARs) for 65–74 years were used [39]; ^‡^ Consumers of fortified foods were defined as those who consumed foods fortified with B-vitamins at least once per week; ^§^ Reference ranges based on analytical laboratory where assay was performed. Abbreviations: BMI, body mass index; MMSE, mini mental state examination; HoloTC, Holotranscobalamin—functional indicator of metabolically active fraction of vitamin B12; MMA, methylmalonic acid—an indicator of vitamin B12 status, a higher MMA status indicates a lower vitamin B12 status; PLP, Pyridoxal-5-phosphate—a measure of vitamin B6 status; EGRAC, Erythrocyte glutathione reductase activation coefficient—a functional indicator of riboflavin status, a higher ratio indicates a lower riboflavin status

**Table 2:** Cognitive Characteristics of healthy older adults at initial examination and after 4-year follow-up (*n* = 155).

	Initial Assessment	Follow-Up Assessment	*p-*Value
Age	69.5 (7.2)	73.4 (7.1)	<0.001
**Cognitive Function Score**			
MMSE Total Score	29.1 (1.3)	27.5 (2.4)	<0.001
Orientation	9.9 (0.3)	9.8 (0.7)	0.014
Registration	3.0 (0.1)	3.0 (0.1)	0.565
Attention	4.7 (0.7)	4.4 (1.1)	0.004
Recall	2.7 (0.6)	1.8 (1.0)	<0.001
Total Verbal	20.3 (1.1)	19.0 (2.0)	<0.001
Language	8.8 (0.5)	8.5 (0.8)	<0.001
Impaired Cognition *n* (%) *	0 (0)	19 (12)	

Data presented as mean (standard deviation) unless otherwise indicated. * Impaired cognition defined as an MMSE score <25 [30]. Differences between the two time points were assessed using a paired *t*-test. *p* ≤ 0.05 considered statistically significant. Abbreviations: MMSE, Mini mental state examination.

**Table 3:** Lifestyle factors, B-vitamin dietary intake and B-vitamin biomarker status as predictors of cognitive decline in older adults.

	Range	Odds Ratio	95% CI	*p*-Value
Age		1.11	(1.05–1.16)	<0.001
Female Gender		0.69	(0.34–1.41)	0.310
BMI		1.04	(0.95–1.13)	0.410
Smoking		2.82	(0.55–14.56)	0.216
*MTHFR* TT genotype		1.82	(0.56–5.93)	0.318
Secondary level education		1.37	(0.62–3.03)	0.434
Depression		0.40	(0.08–2.18)	0.293
**B-Vitamin Biomarker Status**				
Low folate status (RBC Folate) *	(191–719 nmol/L)	1.81	(0.83–3.91)	0.134
Low vitamin B12 (serum total B12) *	(118–231 pmol/L)	1.14	(0.52–2.49)	0.750
Low vitamin B6 (PLP) *	(15.4–42.9 nmol/L)	3.49	(1.60–7.62)	0.002
Low riboflavin status (EGRAC) ^†^	≥1.3	1.01	(0.48–2.15)	0.972
High homocysteine	(12.6–25.4 µmol/L)	1.50	(0.58–3.85)	0.402
**B-Vitamin Dietary Intake ^‡^**				
Low Folate intake	(124–166 µg/day)	2.55	(0.78–8.41)	0.123
Low vitamin B12 intake	(1.2–1.8 µg/day)	1.04	(0.29–3.78)	0.949
Low vitamin B6 intake	(0.9–1.4 mg/day)	4.08	(1.24–13.50)	0.021
Low riboflavin intake	0.6–1.0 mg/day)	0.41	(0.13–1.32)	0.136

Logistic regression was performed to determine predictors of cognitive decline (defined as a decrease in MMSE ≥ 0.56 points/year). The reference category for the lifestyle variables were as follows; sex, male gender; education, 3rd level; depression, no history; MTHFR 677 genotype, MTHFR 677 CC and CT genotype combined. * ‘Low’ B-vitamin status (with the exception of riboflavin) was defined as the bottom tertile of biomarkers; the reference category was the top two tertiles. ^†^ ‘Low’ riboflavin was defined by established cut-off values for EGRAC (low ≥1.3), the reference category was EGRAC < 1.3. ^‡^ ‘Low’ dietary intakes were identified by the bottom 10% of intake for each nutrient, the reference category was the remaining intake. Abbreviations: BMI, body mass index; MTHFR, methylenetetrahydrofolate; RCF, red cell folate; PLP, Pyridoxal-5-phosphate; EGRAC, Erythrocyte glutathione reductase activation coefficient.

## Abbreviations

MMSE	Mini-mental state examination
HoloTC	Holotranscobalamin—functional indicator of metabolically active fraction of vitamin B12
MMA	methymalonic acid—an indicator of vitamin B12 status, a higher MMA status indicates a lower vitamin B12 status
PLP	Pyridoxal-5-phosphate—a measure of vitamin B6 status
EGRAC	Erythrocyte glutathione reductase activation coefficient—a functional indicator of riboflavin status, a higher ratio indicates a lower riboflavin status

## Appendix A

**Table A1:** A comparison of baseline characteristics between participants and non-participants (i.e., those lost to follow-up).

	Participants (*n* = 155)	Non-Participants (*n* = 100)	*p*-Value
**General Characteristics**			
Age (years)	69.5 (7.3)	72.2 (8.1)	0.007
Male *n* (%)	60 (39)	34 (34)	0.530
BMI (kg/m^2^)	27.5 (4.2)	27.3 (3.5)	0.981
Smokers *n* (%)	6 (4)	5 (5)	
**Cognitive Function Score**			
MMSE Total Score	29.1 (1.3)	28.7 (1.4)	0.093
Orientation	9.9 (0.3)	9.8 (0.5)	0.140
Registration	3.0 (0.1)	3.0 (0.1)	0.273
Attention	4.7 (0.7)	4.6 (0.9)	0.243
Recall	2.7 (0.6)	2.6 (0.6)	0.825
Total Verbal	20.0 (1.1)	20.0 (1.2)	0.263
Language	8.8 (0.5)	8.6 (0.6)	0.094
**B-vitamin Biomarker Status**			
Red blood cell folate (nmol/L)	954 (410)	851 (359)	0.080
Serum total vitamin B12 (pmol/L)	282 (106)	257 (127)	0.013
Serum HoloTC (pmol/L)	50.8 (24.3)	47.1 (28.8)	0.381
Serum MMA (µmol/L)	0.24 (0.19)	0.36 (0.56)	0.035
Vitamin B6 (Plasma PLP; nmol/L)	58.4 (25.8)	54.3 (22.9)	0.314
Riboflavin (EGRAC)	1.33 (0.14)	1.34 (0.15)	0.387
Plasma total homocysteine (µmol/L)	12.0 (3.7)	13.1 (4.4)	0.117
**Gastric Function**			
Pepsinogen I (µg/L)	126.8 (70.8)	135.3 (78.2)	0.515
Pepsinogen Ratio^2^	8.4 (6.7)	8.0 (6.6)	0.713

Values represented as mean (SD). Differences in baseline characteristics between those that participated in the 4-year follow-up and those that did not participate in the follow-up were compared using one-way ANCOVA with adjustment for age for continuous variables (on log transformed data were appropriate). Differences in categorical variables were assessed using Chi-squared analysis. *p* < 0.05 was significant.
